# Managing patients on extracorporeal membrane oxygenation support during the COVID-19 pandemic – a proposal for a nursing standard operating procedure

**DOI:** 10.1186/s12912-021-00736-7

**Published:** 2021-10-30

**Authors:** Mateusz Puslecki, Marek Dabrowski, Konrad Baumgart, Marcin Ligowski, Agata Dabrowska, Piotr Ziemak, Sebastian Stefaniak, Lukasz Szarpak, Tammy Friedrich, Lidia Szlanga, Paulina Skorupa, Aleksandra Steliga, Kazimiera Hebel, Blazej Andrejanczyk, Malgorzata Ladzinska, Magdalena Wieczorek, Lukasz Puslecki, Jacek Smereka, Monika Tukacs, Justyna Swol, Marek Jemielity, Bartlomiej Perek

**Affiliations:** 1 Department of Cardiac Surgery and Transplantology, Poznan University of Medical Sciences, Poznan, Poland; 2 Department of Medical Rescue, Poznan University of Medical Sciences, Poznan, Poland; 3 Department of Medical Education, Poznan University of Medical Sciences, Poznan, Poland; 4 Polish Society of Medical Simulation, Poznan, Poland; 5 Sklodowska-Curie Medical Academy, Warsaw, Poland; 6 Polish Society of Disaster Medicine, Wroclaw, Poland; 7 Mayo Clinic, Rochester, MN USA; 8 Department of Thoracic Surgery, Poznan University of Medical Sciences, Poznan, Poland; 9 Department of Cardioanesthesia and Intensive Care, Medical University of Silesia, Zabrze, Poland; 10 Chair of Nursing and Medical Rescue, Pomeranian Academy, Slupsk, Poland; 11 Department of International Management, Poznań University of Economics and Business, Poznan, Poland; 12 Department of Medical Rescue, Wroclaw Medical University, Wroclaw, Poland; 13 Department of Cardiothoracic Intensive Care Unit/Nursing, New York Presbyterian - Columbia University Irving Medical Center, New York, NY USA; 14 Department of Respiratory Medicine, Allergology and Sleep Medicine, Intensive Care Unit, University Hospital of Paracelsus Medical University, Nuremberg, Germany

**Keywords:** Extracorporeal membrane oxygenation, ECMO, Simulation, COVID-19, Nursing, Pandemic

## Abstract

**Background:**

Extracorporeal membrane oxygenation (ECMO) is effective in a selected critically ill patient population with promising results in refractory hypoxemia related to the novel coronavirus disease (COVID-19). However, it requires specialized clinicians and resources in advanced technology. Moreover, the COVID-19 remains an ongoing global emergency, and there is no evidence-based practice in preparedness. This article proposes an innovative and optimized nursing care protocol, the Standard Operating Procedure (SOP), that regulates safety and efficiency in using personal protective equipment (PPE) during ECMO-relevant procedures while providing ECMO therapy for patients with COVID-19.

**Methods:**

After performing a narrative literature search, we developed a high-fidelity translational simulation scenario. It included practicing appropriate donning and doffing PPE during work organization, ECMO-related procedures, and routine daily nursing care and management of ECMO over nine hours. In addition, we held supplementary constructive debrief meetings to consult international expert in the field.

**Results:**

A proposal for nursing standardized operating procedures was created, divided into categories. They included work organization, workload references, competences, infrastructural conditions, cannulation equipment, daily routine nursing care, and procedures during ECMO.

**Conclusions:**

High-fidelity medical simulation can play an important role in staff training, improvement in previously gained proficiency, and development of optimal SOP for nursing care and management during ECMO in patients with COVID-19. Optimal SOPs may further guide multidisciplinary teams, including intensive care units and interventional departments.

**Supplementary Information:**

The online version contains supplementary material available at 10.1186/s12912-021-00736-7.

## Background

A few publications focus on extracorporeal membrane oxygenation (ECMO) and nursing [1–3], with formal guidelines provided by the Extracorporeal Life Support Organization (ELSO) [4]. General standards on the provision of ECMO during the global coronavirus disease 2019 (COVID-19) pandemic are also provided [5]. The dynamic evolution of the pandemic enabled growth for innovations to optimizations of procedures [6–12]. However, no guidelines are specific to nursing care and clinical management of ECMO and the use of personal protective equipment (PPE) during a disaster state such as the current pandemic, leaving ECMO centres to develop their institutional procedures.

Extracorporeal membrane oxygenation can help a selected critically ill patient population [13] in modern critical care. However, its implementation in pandemic conditions is only possible with appropriate human and non-human technological resources [14, 15]. Due to its invasiveness, ECMO is a high-risk application with potential sudden and severe complications such as air entrapment in the ECMO circuit, and dislodgement of cannulae [5, 16]. Accordingly, the constant presence of at least two ECMO-trained clinicians is necessary for emergent life-saving interventions.

From December 2019 to March 2020, COVID-19 evolved from a cluster of pneumonia cases in China into the first coronavirus-caused pandemic [17], surpassing 30.5 million cases and 950,000 deaths in nine months and remaining uncontrolled [18]. It has been accepted that the COVID-19 virus transmits human-to-human through respiratory droplets by coughing, sneezing, and aerosols [11]. The disease is highly contagious, with a relatively long incubation period, an asymptomatic state, and viral shedding post-recovery. The lack of effective antiviral treatment and vaccine in the first month after pandemic outbreak left prevention and supportive therapies the only potentially helpful strategies available at the time. Although estimates are that only 3–5% of all cases progress into critical illness [19], it is significant given the very high numerator. Invasive mechanical ventilation is necessary for a substantial number of COVID 19-cases in both hospitalized and critically ill (2.3–33.1% and 29.1–89.9%, respectively) [12]. The World Health Organization (WHO) provisionally recommended ECMO for cases with refractory hypoxemia unresponsive to lung-protective ventilation, emphasizing *access to expertise in extracorporeal membrane oxygenation* [20].

To apply ECMO in critically ill patients with COVID-19, a careful analysis of the balance between the potential benefits of ECMO and the available ECMO-trained human resources, staff safety, epidemiological restrictions, and hospital equipment and infrastructure is necessary [14, 15, 21]. Currently, there are 2571 applications of ECMO in COVID-19 reported into the Extracorporeal Life Support Organization (ELSO) registry [22].

A translational simulation recently described that integrates innovation, knowledge, skills, and interpersonal collaboration and has proven to be helpful in the introduction of challenging and complex procedures [23–25]. Its value expands to coronavirus pandemic conditions. It involved a collaborative effort in improving system outcomes through identifying potential obstacles and performance issues and implementing the appropriate intervention, irrespective of the location, modality, or circumstances. By functional alignment and quality improvement in dedicated institutions, it encompasses educational interventions targeting practice and outcomes.

### Aim

The purpose of this paper was to develop a proposal for a nursing Standard Operating Procedure applicable to ECMO patients with COVID-19 and generalizable to similar conditions (epidemic, pandemic, disaster, etc.) and all medical professionals involved in extracorporeal life support therapy.

### Area of implementation

This proposal can help standardize practice for medical and nursing clinicians caring for patients with COVID-19 on ECMO.

## Methods

### Narrative literature research

Three investigators (MP, KB, MD) performed a literature search of original papers published by June 2020. Two search engines, PubMed and Google Scholar were used, with the following MeSH (Medical Subject Headings) and Booleans keyword’s: ((“Extracorporeal Membrane Oxygenation”[MeSH] OR “ECMO”[MeSH]) AND (“COVID”[MeSH] OR “coronavirus”[MeSH])) AND “nursing”[MeSH Terms].

### Translational simulation

On a single day, over 12 h, of which three were used for preparation and nine to run the scenarios, the authors carried out the simulation practice in an isolated ICU room in the Centre of Medical Simulation, Poznan University of Medical Sciences.

The simulation was designed and ran by two trainers, two experienced intensive care nurses, one perfusionist, and two physicians (an intensivist and a cardiac surgeon). They all had prior training and experience in ECMO and over ten years of experience in simulation-based education.

**Fig. 1:**
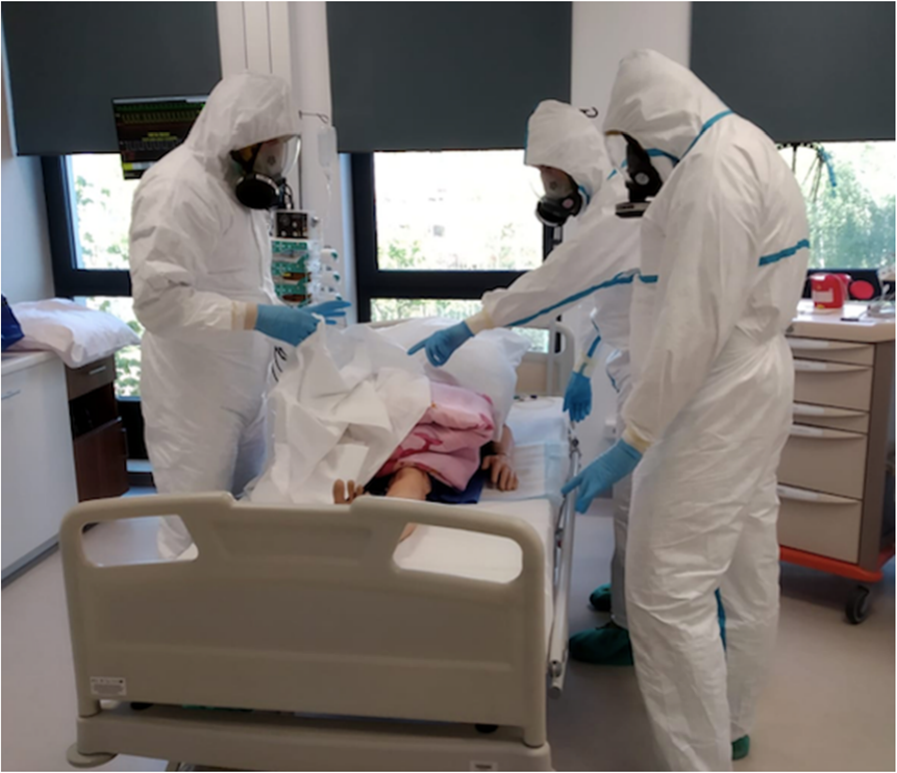
Preparation for ECMO cannulation. ECMO Team in PPE in the isolated ICU room. *(authors’ material)*

The simulation was carried out using a high-fidelity Laerdal 3G SimMan (Laerdal Medical, Orpington, UK) with an incorporated artificial vessel loop in an isolation room in a high-fidelity simulated intensive care unit (ICU) (Fig. 1). Additional equipment used included an invasive mechanical ventilator and a vital sign monitor, infusion pumps, and an ECMO device (Cardiohelp) with a complete ECMO setup of introducers, cannulae, and an HLS Set Advanced set (Maquet, Getinge, Rastatt, Germany). Participants communicated using a radio station (Baofeng – 888S (BaofengTech, USA).

Because this simulation was developed as translational or probing towards creating a nursing protocol for ECMO during COVID-19, therefore its logistics lack the typical structure of a medical simulation. We designed a scenario (Table 1) to test the operational readiness of the ICU environment and staff by monitoring correct PPE use during the care of an ECMO patient. The scenario included a nine-hour simulation of routine work in an ICU with two nurses changing in the non-contaminated (i.e., cold zone) and contaminated area (i.e., hot zone) and a three-hour nursing care “shift” (intervals) in working period time. Additional participants included five medical staff members and two simulation trainers.

**Table 1:** Summary of the simulation scenario

SCENARIO	
SETUP	An intubated patient with COVID-19 in the ICU isolation room
**PROBLEM**	Despite proper treatment and previous prone clinical status does not improve
**TASK**	The team needs to be able to: A. implement VV ECMO B. take care of patient to fulfil the 6-h nursing period including personnel change between “cold and hot zones” C. daily nurse routine activity including ECMO therapy monitoring D. intrahospital transportation E. other actions during ECMO support

During the simulations, participants were required to identify the obstacle related to COVID-19 isolation requirements in taking care of the patient on ECMO. After the scenario, they joined a debriefing session lead by the two simulation trainers. The debrief consisted of a discussion, the development of a checklist, and a proposal of a daily nursing routine. Scenario participants were guided to discuss clinical elements of their experience, including retrospective identification of potential technical problems, hidden risks, equipment failures and alarms, and difficulties in using PPE. Issues regarding human factors were also discussed including personal fears, communication, and teamwork. Then each participant was asked to create an equipment checklist, followed by a proposal for an SOP in ECMO for conditions with isolation requirements specific to COVID-19. The process was arranged in the following steps:

1. Translational simulation stage

- Identification of the problem (PPE, communication, documentation, nursing time for direct patient care, cognitive and behavioral skills, facilitation, and roles of staff members)
- Solution of the problem
- Training

2. Discussion stage

- Debriefing and consultation
- Recommendation
- Proposal for innovation
- SOP proposal

3. Debriefing and consultations

### Debriefing and consultations

We included five participants in the two-hour preparation of Stage 1: developing scenarios, selecting clinical challenges, and identifying solutions. Based on evidence and our experience, translational simulation, and expert consultations, we developed innovations in three main areas, (A) work organization, (B) daily nursing care and management of patients on ECMO and COVID-19, and (C) ECMO cannulation and related procedures. In addition, with the assistance of international experts in ECMO and critical care nursing, we made significant revisions to the initial SOP used at our institution. We invited eleven professionals considered experts in the field. Each had over ten years of experience (including over five in ECMO) from eight different departments. By collaboration, consultation, recommendation, and innovation, they represented expert consensus. They were responsible for Stages 2 of the SOP development.

There was no statistical analysis planned or performed, and all data were qualitatively assessed.

### Ethics

Complying with the requirements of the Local Bioethical Committee of Poznan University of Medical Sciences, since the structure of this simulation did not involve patients, no ethical approval was sought.

## Results

Several publications on ECMO and nursing were identified, mainly recommendations from regional centers [1–3, 26–28]. There ELSO also provides guideline. However, they are limited to the use of PPE during ECMO in patients with COVID-19 [5, 10, 13]. We identified one study dedicated to the nursing management of patients with COVID-19 supported on ECMO [8]. It inlcuded a preliminary outline of the issues, mainly concerning the management of human resources on providing ECMO during the pandemic.

Developing the SOP consisted of indicating main issues during simulated ICU work (Stage 1), constructive debriefing and expert consultation (Stage 2). Our team identified over 20 challenges during probing simulation related to PPE, workload, and non-technical skills. Moreover, this simulation helped identify subtle risks and suggest corrective actions.

The most important aspects that can affect the nursing management of patients on ECMO were noted in non-technical skills. They included dedicated training with in-situ simulation, the use of checklists, and trust among the team members. For more effective communication, wearing a tag with a name and role was suggested.

Participants noted that the lack of a dedicated ECMO specialist for managing the ECMO device creates an additional strain on nursing staff in expertise and time management. Checklists for procedures and use of equipment are necessary to develop based on institutional conditions.

One of the most unexpected findings from the discussion phase was the possibility of the disqualification of a patient previously identified as a potential candidate for extracorporeal support and after all equipment had been collected in a hot zone. At the briefing and expert consultation, it was suggested that to prevent unnecessary contamination of the equipment, all equipment should eventually be moved to the hot area after the patient’s qualification has been confirmed.

**Fig. 2:**
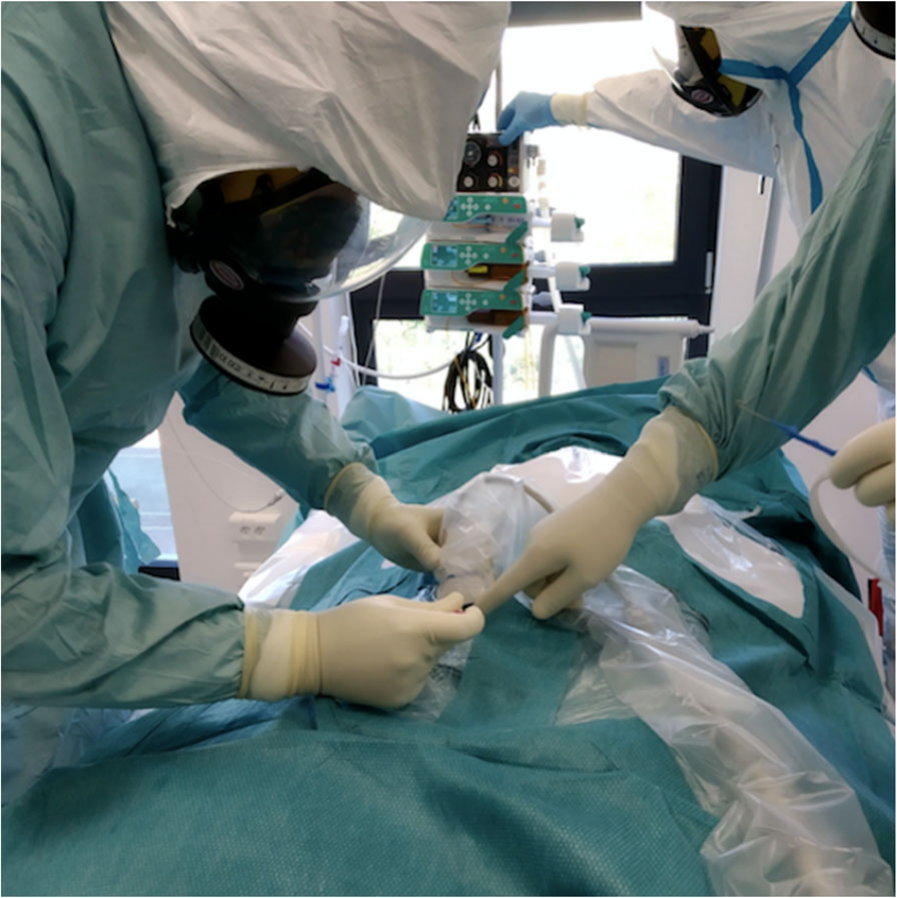
Venous femoral cannulation, percutaneous ultrasound navigated. *(authors’ material)*

The results of the participants’ proposals based on the simulated ICU work are summarized in Table 2 and combined into a final SOP (Stage 2) covering three different areas, including work organization (Appendices A), management, and care of patients on ECMO with COVID-19 (Appendices B), and ECMO cannulation and other ECMO-related procedures (Appendices C) (Fig. 2).

**Table 2:** Results summary of translational simulation, debriefing and expert consultation in management with ECMO supported patients SOP development

Issue	Training	Discussion		Sop
**Translational Simulation**		**Debriefing and consultation** **recommendation**	**Innovation Proposals**	**Appendicities**
**PROTECTIVE EQUIPMENT**				
face masks	• full mask central • full mask lateral	communication difficulties – both lateral – better visibility	proper PPE adopted to local conditions and equipment	A/C
protective clothes	WHO recommendation PPE wear Training	warm discomfort	personnel reduction; shift duration – 3 h; for short intervention – gown acceptable; personnel training in PPE	A/C
**COMMUNICATION/TECHNICALIA**				
radio	radio	communication between zones	adopted to local conditions – in isolated room or multi patients’ room	A/C
mobile phone	hot and cold zone mobile phone	communication between zones	adopted to local conditions – in isolated room or multi patients’ room	A/C
writing on window	writing marker on glass	dedicated “window” dedicated whiteboard	adopted to local conditions – in isolated room or multi patients’ room	A/C
**MEDICAL DOCUMENTATION**				
double documentation	double documentation in cold and hot zone	main problems written on whiteboard	part of documentation printed from hot zone on cold zone printer	B/C
paper/electronic	paper and electronic	a lot of paper waste	electronic documentation in hot zone, except medical orders	B/C
**WORKING TIME AND NURSE-PATIENT RELATION**				
working time	proposed 3–6 h shift	optimized working time should not exceed 3 h in hot zone and 3 h in cold zone during 12 h duty	proposed personnel changes for every 3 h	A/C
staff optimization and exposure	• nurse-ECMO patient relation during care - 1:1 • ECMO implantation in 3 persons	in wards overloading accepted relation – 1:2 for patient safety ECMO implantation should be performed in min 2 persons	• proper checklists for equipment: CPR or ECMO implantation should be performed • proper checklists for minimal personnel activity during special interventions should be prepared • nurse-ECMO patient relation during care is depend to local conditions	B/C A/C A/C
ECMO competences in nursing personnel	• ECMO specialist available • ECMO specialist not available	• circuit check is performed every 12 h • circuit check should be included into nursing daily routine	• Training in basic skills of operating the ECMO system: assessment of system integrity, possible complications (formation of thromboembolic material, air embolism, presence of leaks) and ensuring safety when changing patient’s position. • Critical conditions requiring immediate reaction should be identified (air, line disconnection, no power and/or oxygen supply). • Protocol for indications for interventional line clamping - on-the-job training (accidental decannulation, pump failure, air embolism).	A/B/C
				A/B/C
				A/B/C
patient’s identification	adopted to local protocol; visible bed number	• resignation from sensitive data • adopted to local conditions and legislation	• patient names in nursing area placed on whiteboard – doubled in cold zone • patients’ bed with numbers monitored by industrial cameras	A
**COGNITIVE AND BEHAVIORAL SKILLS** **Non-technical skills**				
competence	• team consists of leader in every cases • variety roles	in the case of a small number of ECMO specialists, the nurse must take care of circuit device.	• dedicated training for different expectations • ECMO management is part of nursing daily routine	B/C B
trust in a team mutual respect	• the staff knows each other • group of people working together in “normal” conditions	• 3 h shifts – rotation induces an unfamiliarity between the staff • PPE makes it difficult to recognize visually people/roles	• team prebriefing • teamwork – Team responsibility • team support • names label/writing names and role on back of full apron • only written orders should be prepared	–
knowledge sharing	not ignoring messages	think at loud and inform team members	every signal about new condition should be checked	–
knowledge about own limitations	fear of letting me know/don’t know something	• every team member knows own limitation • masking restrictions can be critical	personnel checklist of procedures/interventions and competency	–
close loop communications	• double confirmation about closes a task (orders) • PPE (full face mask) limited voice and it leads to confusion • difficult listening to orders	• eye contact (direct confirmation) decreased misunderstanding • no written orders - life threatening condition	• minimizing the risk of error - preferably the execution of only saved orders and full confirmation • the principle of trust • checklists - to be confirmed • equipment checklist	- B/C B/C
guidelines in.eg. CPR or sudden interventions	the staff is trained	when hustle and bustle and disorder appear low-quality of interventions	• continuous training in different conditions – in various professional groups • in situ simulation (own environments) • training in hot zone conditions	- - -
**FACILITATION**				
orders checklists	a designated time frame for medical orders	closed-loop in communication	only written orders should be prepared	–
equipment checklists	prepared protocols/checklists for CPR, ECMO implantation	minimizes the risk of being forgotten or overlooked	centers should prepare own checklists for equipment and staff especially for separated hot zone and different for dedicated departments or temporary hospitals	A/B/C
**PERSONNEL ROLES**				
personality	problems with personnel recognition and communication	staff in PPE to wear labels (marker pen on tape) with their name and role on their visor	writing names and role on back of full apron	A
new roles	nurse new skills	assistance in extracorporeal support preparation for proper interventions	checklists for new activities protocols in individual Teams for interventions ECMO circuit check included in Nursing Daily Routine	C C B
leader	prepared for leading role	leader in cold zone, prepared for intervention in hot zone	• responsible for local checklists for personnel activity and interventions preparation	–
medical orders	in written form	in written form allow to eliminate communication orders	• only written orders should be prepared, especially pharmacotherapy • except emergency interventions – in. ex. CPR	- -
**LATENT HAZARDS AND CORRECTIVE ACTIVITY EXAMPLES**				
lack of comfort with PPE	• lack of comfort PPE - 4 gloves for aseptic during cannulation • masks & gowns led to staff becoming overheated and uncomfortable	• only 3 pair gloves are necessary – last sterile • minimalization of staff and shift limit to 3 h	• 3 pair gloves are recommended • staff optimization	- A
needs for hot and cold zone preparation	in situation when separated room or department hot zone is separated equipment should be doubled	equipment duplication in Airway / Ventilation / Circulation / Extracorporeal	optimization and rationalization of equipment	C
risk unnecessary contamination of equipment	rationalization of the additional equipment disposal in. eg. ECMO, USG	in case of disqualification from implantation ECMO when ECMO Team went to hot zone with equipment - risk of contamination	final equipment collection after final qualification of the patient by the implantation team	–
organizational culture of logistics	unnecessary additional relocation of the staff and equipment	identification of the amount of equipment needed	centers should prepare own checklists for equipment and staff especially for separated hot zone and different for dedicated departments or temporary hospitals	A/C
situationalawareness	Loner time for staff and equipment preparation	lack of knowledge and practice	PPE wearing training and routine function with PPE	A
unreadable messages in the lock	risk of contamination during PPE uncovering	recommended preparation of legible messages and labels with the use of pictograms	• proper training • high fidelity medical simulation • translational simulation	–
risk anxiety due to lack of training and familiarity	important in new personnel redistribution	directing inexperienced staff is unavoidable	• proper training • high fidelity medical simulation • translational simulation	–

## Discussion

A simulation is an excellent tool to assess and reassess skills, knowledge, and technical circumstances. Previously, we described our use of high-fidelity simulations to creating protocols for infrequent, new, and complex procedures in the form of translational simulation [1]. Similarly, simulation can be used to develop standards for new procedures specific to ECMO in two ways, (I) an introduction to develop a procedure or a program with initial teaching for newly starting ECMO programs, or (II) a validation tool for ECMO competency based on real-experience scenarios for established ECMO programs. In our case, it was the former, inevitably lacking a typical structure of the latter (e.g., medical simulation, knowledge evaluation of participating clinical staff before and after simulation, debriefing). However, it was essential for developing an ECMO program at a regional ECMO center. We provide recommendations in the Appendices (A, B, and C) focusing on all aspects of nursing care, including underlying discrepancies and innovations. We believe no general discussion is necessary for SOPs prepared this way.

Translational simulation is an effective probing tool in crisis conditions such as the COVID-19 pandemic. The Medical Simulation Center needs to be a part of disseminating knowledge and education. The COVID-19 pandemic created a global crisis in healthcare, forcing changes I established standards of practice in terms of using PPE and general isolation precautions. (re) training clinicians, reorganizing existing medical infrastructure and facilities, and creating new ones.

Additionally, it is possible to recognize issues through reports and data analysis regarding COVID-19 infections. Evolving challenges influencing the healthcare sector like viral mutations and vaccine development targeting those call for new collaborations and knowledge sharing among different stakeholders in healthcare, helping to defeat COVID-19 pandemic faster [29]. New trends are published in professional journals in medicine, economics, management, informatics technology, etc., on analyzing the impact of the COVID-19 pandemic from different perspectives. Collaboration of this expanded multi-professional group is necessary for developing and carrying out effective plans. The same in clinical areas of patient care is important using modern technology (digital Health, medHealth, telehealth, telemedicine, personalized medicine, etc.) for solutions in protecting healthcare personnel, quickly detect and prevent the spread of COVID-19, and improve intensive care for the patient [29]. The R&D cooperation between academia, institutions, and businesses, with the use of advanced knowledge and technology, can also significantly contribute to improve medical therapy (e.g., design and manufacture safe vaccines against COVID-19).

Typically, the lack of the possibility for clinical training of medical personnel is balanced by using medical simulation. Medical centers worldwide found themselves in the same situation providing training for clinicians most exposed to the COVID-19 infection. The pandemic also necessitated changes in critical care. In these difficult times, we need to improve cooperation and use of local potential in collaboration with partners to develop better therapies for patients [29]. The knowledge hub in the ecosystem of medical simulation, responsible for integrating external partners, coordinating innovative cooperation, transferring and diffusing knowledge, monitoring, managing, and controlling its’ function, continuously improving existing procedures and qualifications and skills of professionals within a dedicated educational platform is essential [30]. By getting involved in cooperation and sharing knowledge and experience through patient-centered care and innovation, we can contribute to a more rapid defeat of the pandemic [30, 31].

### Appropriate level of PPE

The PPE used by personnel working in the “hot zone” during routine care was defined by the local epidemiological institutional policies and the availability of PPE.

**Fig. 3:**
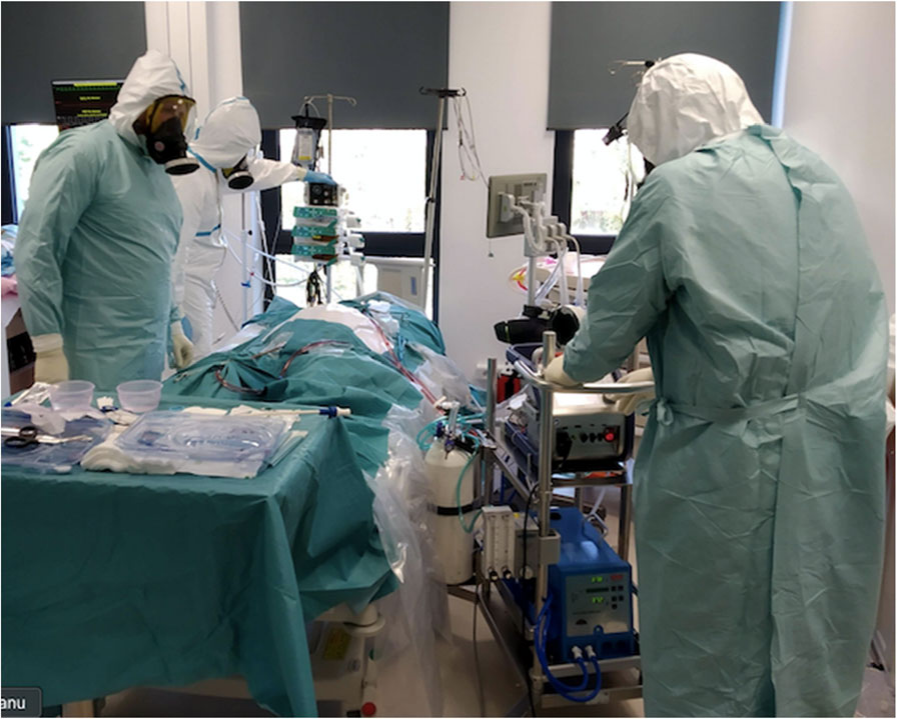
VV ECMO run in isolated ICU room. *(authors’ material)*

**Fig. 4:**
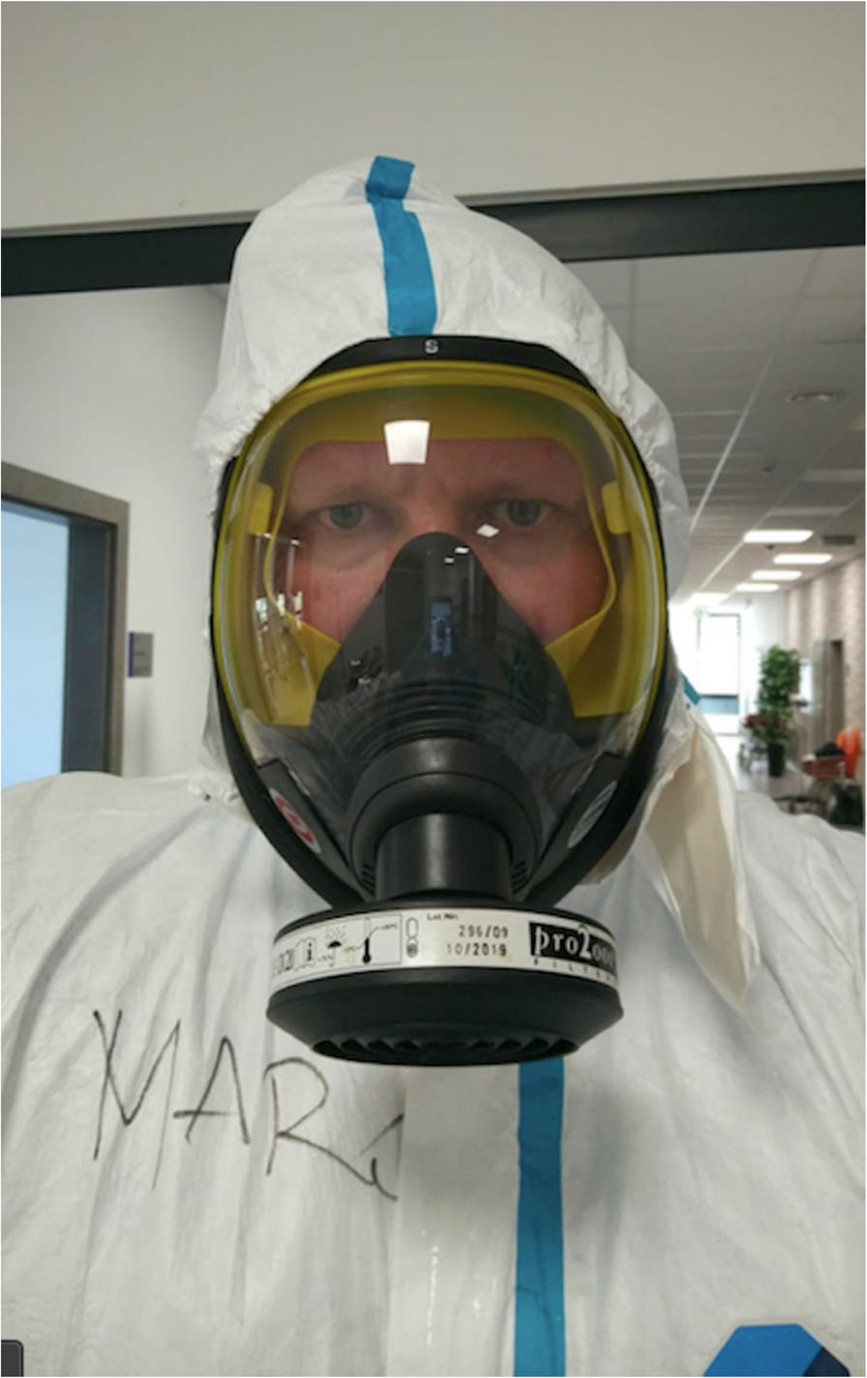
Full-face mask with absorber (Scott-Vision 4000 with Pro2000 filter, 3 M, USA). Personnel name marked on apron. *(authors’ material)*

The WHO provides guidelines for both prevention and protection related to COVID-19. Studies demonstrated that for performing aerosol-generating procedures, the proper level of PPE includes a respirator, eye protection goggles, a gown with long sleeves, and a full-length isolation gown [7, 8, 20, 28]. The correct use of PPE by healthcare professionals is elemental for limiting the spread of infection. Detailed recommendations for the using PPE during ECMO have been included in the latest COVID-19 guidelines by ELSO [5]. A full-face mask with an absorber and a level 3 protection (Scott-Vision 4000 with Pro2000 filter, 3 M, USA) is safe and comfortable (Fig. 4). A full-face mask prevents evaporation and does not decrease voice reception. With horizontal and vertical curvatures and excellent optical properties, it does not distort the wearer’s view. Our simulation demonstrated better protection by full-face masks with a side filter connector than a bottom (chin) mask, as it covers and sits on the front of the protective clothing (Figs. 3 and 4).

The layers of PPE covering the clinician can be a source of miscommunication in contaminated areas, so our team suggests increasing the use of non-verbal communication methods such as writing, eye contact, and closed-loop communication. To decrease stress in actual clinical areas, we highly recommend in-situ simulation wearing PPE.

### Critical care in an isolation room

In many ECMO centers, the criteria for ECMO have been restricted due to a lack of human resources. Our simulation has demonstrated two key factors important for managing patients with COVID-19, the ability to properly communicate and strategically minimize the number of clinicians in the patient’s room. The balance between providing an efficient therapy and safety of personnel requires strict control with PPE, compliance with respecting the hot and cold zones [8], and adherence to standards of involving only the necessary number of clinicians in the isolation room [20, 32].

Lessons learned from epidemics and pandemics of the past are incorporated into contemporary strategies of modified systems including timely notifications, isolation precautions, infection prevention, and environmental cleanliness of ICUs [6, 8, 11, 33]. Minimizing the frequency of opening the door of the patient’s room was significant during the simulation. Reducing the nurse-to-patient ratio to 1:1 will be necessary for the aspect of lack of human resources. Additionally, it decreases the frequency the nurse moves in and out of the room [34, 35]. Assigning one nurse for each zone, efficient nursing communication, and a larger isolation room helped improve efficiency in therapy. Exceptions are situations that require the presence of additional clinicians (e.g., CPR, patient prone positioning or transferring). Optimal work time should not exceed three hours in the hot zone and three hours in the cold zone during a 12-h duty, and in the case of a limited number of ECMO specialists, when the nurse must take care of the ECMO device and circuit.

For collecting equipment, using a checklist was a new and efficient approach (Appendices C and Table 3).

**Table 3:** ECMO cannulation equipment checklist proposal

EQUIPMENT	No	
Cardiohelp	1	
HLS set	1	
Hand crank	1	
Clamp	min 4	
Male/male Luer Lock connector	min 2	
Tap with extension	min 8	
SHUNT 6-7Fr	min 2	
Introducer set with multi-step dilators and wires	2 × 100 cm; 2 × 150 cm	
Venous cannula 23Fr, 25Fr	2 × 2	
Arterial cannula 17Fr, 19Fr	2 × 2	
ECMO cover set (with sutures)	1	
Disinfection – BRAUNODERM/CITROCLOREX 2%	1	
PPE	3	
Sterile dressing	min 2	
Infusion fluids - STEROFUNDIN	2 l	
Head light	min 1	
USG device	1	
USG sterile probe cover	2	
Cannula washing set: 100 ml syringe, saline, heparin	1	

### Specific care related to ECMO

Managing a patient with COVID-19 also on ECMO requires additional multidisciplinary staff with expertise in ECMO. Moreover, the unknown of the novel pandemic mandated modifying standards related to the use of PPE and the increased patient workload. Optimization, maintenance, and adherence to isolation specific to COVID-19 are necessary to reduce the risk of transmission of infection to the highly specialized ECMO team. Their well-being is crucial for providing ECMO support to the most critically ill COVID-19 patients [9].

### Pandemic-specific nursing management

In the proposed SOP, nurses play an integral role in the ECMO team. They are responsible for monitoring the ECMO device and patient hemodynamics [10, 36], and responding to ECMO emergencies, while following the strict infection control specific to COVID-19. This can lead to significant physical and mental stress [8]. Ongoing training, including appropriate use of PPE, daily management of ECMO, and readiness for ECMO emergency, helps less experienced clinicians prevent burnout and improve preparedness for any upcoming pandemic [10]. For collecting equipment, direct checklists should be utilized.

### Innovation

It seems reasonable to build isolation rooms with easy viewing, with continuous monitoring and communication ability from outside the room, especially during procedures. An alternative solution can be to design a control room with constant monitoring of the patient with projecting two cameras, at a minimum.

### Communication

We have learned that using PPE required for patients with COVID-19 causes additional anxiety and discomfort to staff. Nursing care and communication among team members is an added challenge [32]. The introduction of facilities over intercom, continuous availability, and assistance of a nurse from the “cold zone”; names written on tags and secured on isolations gowns of the auxiliary staff, writing essential information on a window, glass or board helps decrease stress and inconveniences (Fig. 4). Our simulation also demonstrates the usefulness of full-face masks and wireless radio systems between the zones for communication. Part of communication support can be radio, mobile phone or writing on glass window. In some situations, only written orders should be used, especially for pharmacotherapy.

### Training and simulation

Providing ECMO therapy requires the education and training of clinicians, including high-fidelity simulation [10]. Moreover, due to the current global state of the pandemic, added attention is necessary. Medical simulation in ECMO has been used for almost 20 years [10, 32, 33, 36]; it created new procedures and built ECMO-dedicated teams [16, 37, 38].

When providing ECMO for patients with COVID-19, diligence in caring for the patient, nursing management, and monitoring the ECMO device are fundamentals of nursing. Training nurses to monitor the ECMO circuit, including, but not limited to the colour of blood in the ECMO tubing, pressures in the circuit pre- and post- oxygenator, and interventional clamping of ECMO cannulas in an ECMO emergency [36]. We know from our past simulations that nurses can detect potential emergencies and ad hoc interventions preceding support from “cold zone”. It is significant for training that checklist for collecting equipment, staff activity, interventions, troubleshooting the ECMO device alarms, and routine nursing care are used (Appendices B).

### Limitations

This is a single-center study, hence the implementation of the approach used for the simulation is feasible only at centers with similar structures. Because each hospital’s critical care department has a different organizational structure, the impact of a pandemic state can cause unique conditions, which cannot be objectively created for simulation training purposes. Additionally, our SOP proposals have not been evaluated formally. Given the unprecedented circumstances of COVID-19, such an evaluation was not possible. While this proposal lacks formality, and is not easily generalizable, it is comprehensive, and, to our knowledge, remains the only proposal in this area.

## Conclusion

High-fidelity simulation can enhance existing expertise in clinical standards of care. Although an unprecedented pandemic creates unpredictable barriers, we demonstrated the possibility of implementing an initial simulation strategy resulting in a proposal for a nursing SOP for the most critically ill with COVID-19 on ECMO therapy. This simulation project can guide intensive care units and departments of operations in aspects of care that may require focus and modifications before admitting COVID-19 patients or in a future pandemic. We recommend implementing in situ simulation training to identify unit- or institution-specific risks and create individualized protocols. Other available guidelines along with our proposal are recommended to consider and modify to own unique circumstances. Cooperation and willingness to share knowledge and experience using medical simulations will likely allow developing and improving novel SOPs in increased safety of clinicians and patients with COVID-19 on ECMO support.

### Terms

“cold zone” - epidemiologically clean zone.

“hot zone” - epidemiologically contaminated zone.

## Supplementary Information

**Additional file 1: Appendices A.** Organization of patient care environment. The normal font contains innovation suggestions, italic commentary as integrated part of discussion.

**Additional file 2: Appendices B.** Direct care of COVID-19 patients with ECMO – nurse daily routine activity. The normal font contains innovation suggestions, italic commentary as integrated part of discussion.

**Additional file 3: Appendices C.** Procedures with extracorporeal techniques. The normal font contains innovation suggestions, italic commentary as integrated part of discussion.

## Data Availability

Not applicable.

## Abbreviations

ABM: acid-base management
ACT: activated clotting time
APTT: activated partial thromboplastin time
COVID-19: coronavirus disease
CVP: *central venous pressure*
ECMO: extracorporeal membrane oxygenation
ECMO VA: ECMO venoarterial
ECMO VV: ECMO venovenous
ELSO: Extracorporeal Life Support Organization
ELS: Extracorporeal Life Support
ETT: endotracheal tube
FFP: filtering facepiece
HR: heart rate
MACC: mechanical automated chest compression
MAP: mean arterial pressure
MPC: medical product characteristics
POCT: point of care testing
SARS-CoV-2: Coronavirus
SOP: standard operating procedure
SpO_2_: oxygen saturation
PPE: personal protective equipment
TISS: Therapeutic Intervention Scoring
VAE: ventilator associated events

## References

[1] Calhoun A (2018). Nursing Care of Adults Patients on ECMO. Crit Care Nurs Q.

[2] Kiersbilck CV, Gordon E, Morris D (2016). Ten things that nurses should know about ECMO. Intensive Care Med.

[3] Redaelli S, Zanella A, Milan M, Isgro S, Lucchini A, Pesenti A (2016). Daily nursing care on patients undergoing venous–venous extracorporeal membrane oxygenation: a challenging procedure!. J Artif Organs.

[4] Brogan TV, Lequier L, Lorusso R, MacLaren G, Peek G. The Red Book: the ELSO Red Book. 5th Edition, ELSO; 2017.

[5] Shekar K, Badulak J, Peek G, Boeken U, Dalton HJ, Arora L, Zakhary B, Ramanathan K, Starr J, Akkanti B, Antonini MV, Ogino MT, Raman L, Barret N, Brodie D, Combes A, Lorusso R, MacLaren G, Müller T, Paden M, Pellegrino V, on behalf of the ELSO Guideline Working Group (2020). Extracorporeal life support organization COVID-19 interim guidelines: a consensus document from an international group of interdisciplinary extracorporeal membrane oxygenation providers. ASAIO J.

[6] Litton E, Bucci T, Chavan S, Ho YY, Holley A, Howard G, Huckson S, Kwong P, Millar J, Nguyen N, Secombe P, Ziegenfuss M, Pilcher D (2020). Surge capacity of intensive care units in case of acute increase in demand caused by COVID-19 in Australia. Med J Aust.

[7] Newby JC, Mabry MC, Carlisle BA, Olson DM, Lane BE (2020). Reflections on nursing ingenuity during the COVID-19 pandemic. J Neurosci Nurs.

[8] Umeda A, Sugiki Y (2020). Nursing care for patients with COVID-19 on extracorporeal membrane oxygenation (ECMO) support. Glob Health Med.

[9] Li X, Guo Z, Li B, Zhang X, Tian R, Wu W, et al. Extracorporeal Membrane Oxygenation for Coronavirus Disease 2019 in Shanghai, China. ASAIO J. 2020;(5):66,475–81. 10.1097/MAT.0000000000001172. 10.1097/MAT.0000000000001172 PMC7273861 32243266

[10] Ramanathan K, Antognini D, Combes A, Paden M, Zakhary B, Ogino M, MacLaren G, Brodie D, Shekar K (2020). Planning and provision of ECMO services for severe ARDS during the COVID-19 pandemic and other outbreaks of emerging infectious diseases. Lancet Respir Med.

[11] Lotfi M, Hamblin MR, Rezaei N (2020). COVID-19: transmission, prevention, and potential therapeutic opportunities. Clin Chim Acta.

[12] Wunsch H (2020). (2020). Mechanical ventilation in COVID-19: interpreting the current epidemiology. Am J Respir Crit Care Med.

[13] Combes A, Hajage D, Capellier G, Demoule A, Lavoue S, Guervilly C (2018). Extracorporeal membrane oxygenation for severe acute respiratory distress syndrome. N Engl J Med.

[14] Smereka J, Puslecki M, Ruetzler K, Filipiak KJ, Jaguszewski M, Ladny JR, Szarpak L (2020). Extracorporeal membrane oxygenation in COVID-19. Cardiol J.

[15] Smereka J, Szarpak L, Filipiak KJ (2020). Modern medicine in COVID-19 era. Disaster Emerg Med J.

[16] Puślecki M, Ligowski M, Dąbrowski M (2018). BEST life - "bringing ECMO simulation to life" - how medical simulation improved a regional ECMO program. Artif Organs.

[17] Listings of WHO's response to COVID-19. (n.d.). World Health Organization. Retrieved September 19, 2020, from https://www.who.int/news-room/detail/29-06-2020-covidtimeline

[18] COVID-19 situation update worldwide, as of 17 September 2020. (2020, September 17). European Centre for Disease prevention and control. Retrieved September 19, 2020, from https://www.ecdc.europa.eu/en/geographical-distribution-2019-ncov-cases

[19] Auld SC, Caridi-Scheible M, Blum JM, Robichaux C, Kraft C, Tacob JT (2020). ICU and ventilator mortality among critically ill adults with coronavirus disease 2019. Crit Care Med.

[20] World Health Organization. (2020). Clinical management of COVID-19: interim guidance. 27 May 2020 (No. WHO/2019-nCoV/clinical/2020.5). World Health Organization Global, from https://www.who.int/publications-detail-redirect/clinical-management-of-covid-19

[21] Ruetzler K, Szarpak L, Filipiak KJ, Ladny JR, Smereka J (2020). The COVID-19 pandemic — a view of the current state of the problem. Disaster Emerg Med J..

[22] Extracorporeal Life Support Organization; https://www.elso.org (2020). Accessed 25 Sept 2020.

[23] Brazil V (2017). Translational simulation: not 'where?' but 'why?' A functional view of in situ simulation. Adv Simul.

[24] Abelsson A (2017). Learning through simulation. Disaster Emerg Med J..

[25] Czekajlo M, Dabrowska A (2017). In situ simulation of cardiac arrest. Disaster Emerg Med J..

[26] Seczyńska B (2018). Extracorporeal gas exchange.

[27] Mirabel A, Jehanno AC, David CH, Lebreton G. Preparing the patient and the ECMO device: Mossadegh C, Combes a. Nurs Care ECMO Cham. 2017:39–44. 10.1007/978-3-319-20101-6_4.

[28] Cook TM (2020). Personal protective equipment during the coronavirus disease (COVID) 2019 pandemic – a narrative review. Anaesthesia..

[29] Puslecki L, Dabrowski M, Puslecki M. Development of innovation cooperation in the time of COVID-19 pandemic. Eur Res Stud J. 2021;(Special Issue 1):1049–73. 10.35808/ersj/2087.

[30] Puslecki L, Czekajlo M, Puslecki M. How innovation cooperation supports the improvement of health care in the CEE region: the case of ECMO for greater Poland. Eur Res Stud J. 2021;(Special issue 1):1074–95. 10.35808/ersj/2088.

[31] Puslecki M, Baumgart K, Ligowski M, Dabrowski M, Stefaniak S, Ladzinska M, Goszczynska E, Marcinkowski P, Olasinska-Wisniewska A, Klosiewicz T, Pawlak A, Zielinski M, Puslecki L, Podlewski R, Szarpak L, Jemielity M, Perek B (2021). Patient safety during ECMO transportation: single center experience and literature review. Emerg Med International.

[32] Fregene TE, Nadarajah P, Buckley JF, Bigham S, Nangalia V (2020). Use of in situ simulation to evaluate the operational readiness of a high-consequence infectious disease intensive care unit. Anaesthesia.

[33] Funk DJ, Siddiqui F, Wiebe K, Miller RR, Bautista E, Jimenez E (2010). Practical lessons from the first outbreaks: clinical presentation, obstacles, and management strategies for severe pandemic (pH1N1) 2009 influenza pneumonitis. Crit Care Med.

[34] Mossadegh C. Monitoring the ECMO: Mossadegh C, Combes a. Nursing Care and ECMO. Cham: Springer. 2017:45–70. 10.1007/978-3-319-20101-6_5.

[35] Lucchini A, Elli S, De Felippis C, Greco C, Mulas A, Ricucci P, Fumagalli R, Foti G. The evaluation of nursing workload within an Italian ECMO Centre: a retrospective observational study. Intensive Crit Care Nurs 2019;55:102749. doi: 10.1016/j.iccn.2019.07.008. Epub 2019 Aug 7. PMID: 31400831. 10.1016/j.iccn.2019.07.008 31400831

[36] Mędrzycka-Dąbrowska W, Czyż-Szypenbejl K, Katarzyna K-J, Lewandowska K, Ozga D (2018). Nursing in critical care unit. Pielęgniarstwo w Anestezjologii i Intensywnej Opiece.

[37] Puślecki M, Ligowski M, Stefaniak S, Zieliński M, Pawlak A, Dąbrowski M (2017). Using simulation to create a unique regional ECMO program for the greater Poland region. Qatar Med J.

[38] www.ecmo.pl Accessed 25 Jul 2020.

[39] Padilha KG, Sousa RM (2007). Kimura M, Miyadahira AM, Monteiro da Cruz DAL, de Fatima Vattimo M, et al. nursing workload in intensive care units: a study using the therapeutic intervention scoring System-28 (TISS-28). Intens and Crit Care Nurs.

[40] American Association for Respiratory Care. AARC Clinical Practice Guidelines. Endotracheal suctioning of mechanically ventilated patients with artificial airways 2010. Respir Care 2010;55(6):758–764. PMID: 20507660. 20507660

[41] Techanivate A, Kumwilaisak K, Samranrean S (2008). Estimation of the proper length of orotracheal intubation by Chula formula. J Med Assoc Thail.

