# Supplementary material for: Managing patients on extracorporeal membrane oxygenation support during the COVID-19 pandemic – a proposal for a nursing standard operating procedure
Source: BMC Nurs. 2021 Oct 30;20:214. doi: 10.1186/s12912-021-00736-7 (PMC8556777; doi:10.1186/s12912-021-00736-7)
Supplement: Supplementary file 1 — Additional file 1: Appendices A. Organization of patient care environment. The normal font contains innovation suggestions, italic commentary as integrated part of discussion. [file 12912_2021_736_MOESM1_ESM.docx]

**PART A. ORGANIZATION OF PATIENT CARE ENVIRONMENT**

*The normal font contains innovation suggestions, italic commentary as integrated part of discussion.*

1. **PLACE AND WORKING TIME (including PPE)**
2. **PLACE OF THERAPY** – intensive care unit dedicated to perfusion therapies based on their experience.

*Grouping of patients undergoing ECMO is rational for optimal use of intensive care employees.*

1. **LENGHT OF WORK** – it is proposed to shorten the change time of nurses to 6 hours – based on the period of safe use of the PPE set and the component with the shortest durability. It applies most often to the filtering face piece 3 (FFP3) mask.

It seems rational to divide the team on a given shift into the first 3 hours of work in the “hot zone” followed by change to 3 hours in the “cold zone” and vice versa.

The team in the “cold zone” remains on standby and after the alarm is initiated immediately. Rescue actions should be taken as soon as possible, after nursing staff is secured in PPE.

*Rational personnel management means minimal number of necessary people (difficult working conditions due to high workload, obligation to use PPE, but also rational management with PPE). The staff in „hot zone” stays 3 hours in the room - important preparation before entering (toilet, hydration). Leaving the room is allowed only in justified cases (rational management with PPE) - the need to plan care and activities (accumulation and performance).*

***PPE - the level of protection used by personnel remaining in the “hot zone” during routine care (overalls suit or barrier gown) is determined by the epidemiological procedures locally adopted in the hospital and the availability of PPE.***

***Detailed guidelines for the use of PPE during extracorporeal therapies have been included in the latest ELSO COVID guidelines [5]*.**

*It is necessary to put the staff names on aprons for better communication.*

1. **SYSTEM** **OF** **CHANGES** – the system requires the division of personnel into a "dirty" team secured in PPE for work in a “hot zone” and a "clean" one dedicated to stay in a “cold zone”. The task of the latter personnel is to provide the necessary equipment to the „hot zone”. Efficient communication (radio or intercom) is absolutely mandatory.

*Staff in the “cold zone” reduces the risk of irrational wear of PPE and use of medical equipment. All aforementioned items are delivered on an ongoing basis as needed. They are stored in the safer “cold zone”, and after use in the “hot zone” must be destroyed/utilized or treated in appropriate way.*

1. **WORK LOAD, REFERENCES AND COMPETENCES**
2. **Assignment of roles** – staff working in the „hot” and „cold zone”.

*It is proposed to place the healthy employees in the “hot zone”. High-risk personnel (elderly, with medical history, threatening diseases) should be directed to the “cold” area.*

1. **Nurse-Patient relation** – As a standard, one nurse anaesthetist per ECMO patient (1:1) is required, while, in particular cases to set the device in the FREE mode if one nurse must take care of more than one patient, one nurse per 2 patients (1:2) are recommended [38,42].

***Determine with the therapeutic team what mode the device works:***

***FREE MODE -*** *the device does not stop working despite the alarm being triggered,*

***INTERVENTION MODE -*** *the device stops when the alarm is activated.*

*To objectively determine the workload of nurses, the TISS 28 scale can be used* [2,28].

1. **Qualifications and competences** – ICU nurse/nurse anaesthetist (specialist in anaesthesiologic and intensive care nursing – depends on local regulations).

*Basic skills of operating the ECMO system: assessment of system integrity, possible complications (formation of thromboembolic material, air embolism, presence of leaks) and ensuring safety when changing patient's position* [2,5,29,37,39,42]. *In addition, take into account critical conditions requiring immediate reaction (air, line disconnection, no power and/or oxygen supply). It is important to establish in the local ECMO Team indications for interventional line clamping - on-the-job training (accidental decannulation, pump failure, air embolism).*

1. **INFRASTRUCTURAL CONDITIONS**
2. **Isolation** – an isolation room for a single patient with access through a lock and a vacuum or a multi-person room for patients with COVID on ECMO (lock, vacuum)

*Creating of not only the clear borders between “hot" and "cold zone” but also transition one between them.*

1. **Dressing up** - places designated for PPE dressing and undressing.

*Cloakroom and lock for staff with toilets and showers.*

1. **ONLY NECESSARY** medical equipment and devices are in the patient's room.
2. For individual (local) teams, drug management should be resolved.

*The following issues must be established: a storage of drugs and bedside preparation, who prepared the medicines ("cold' or 'hot" staff) and how they are transfer between zones Institutional and individual decisions should be made after assessing the availability of therapeutic staff and after considering the location of the therapy (a room of isolation or a therapy room for several patients).*
