# Supplementary material for: Managing patients on extracorporeal membrane oxygenation support during the COVID-19 pandemic – a proposal for a nursing standard operating procedure
Source: BMC Nurs. 2021 Oct 30;20:214. doi: 10.1186/s12912-021-00736-7 (PMC8556777; doi:10.1186/s12912-021-00736-7)
Supplement: Supplementary file 2 — Additional file 2: Appendices B. Direct care of COVID-19 patients with ECMO – nurse daily routine activity. The normal font contains innovation suggestions, italic commentary as integrated part of discussion. [file 12912_2021_736_MOESM2_ESM.docx]

**Part B. DIRECT CARE OF COVID-19 PATIENTS WITH ECMO**

**– NURSE DAILY ROUTINE ACTIVITY**

*The normal font contains innovation suggestions, italic commentary as integrated part of discussion.*

It is important to prepare proper nursing staff with competence to take care of a patient in critical condition on ECMO therapy. Apart from the routine actions of intensive care, the additional areas of particular attention include proper stabilization of vascular cannulas, monitoring of the ECMO device and connected circuit, dosing and monitoring of anticoagulation, gas flow; proceedings in the urgent complications, connecting a renal replacement therapy, transporting the patient, medical documentation [2,3,5,26].

**GENERAL CARE RECOMMENDATION:**

- On the ECMO console there is an emergency number posted for a perfusionist or ECMO physician available 24/7.
- The monitoring is typical for a patient in the ICU with a 'head-to-foot' rating [3]:
- vital signs: HR (heart rate), MAP (mean arterial pressure), SpO2 (oxygen saturation), patient temperature, CVP (central venous pressure),
- physical examination (features of hypoperfusion, sweating, degree of hydration), stool excretion and measurement of diuresis on hour basis,
- neurological status (state of consciousness, response to nursing activities, reaction of pupils to light),
- control of all vascular accesses, monitoring of the ECMO system and prevention of potential complications.
- Planning all care activities and procedures. Assembly of the necessary equipment. Assigning roles to all team members - if possible, marking them on the suit in a visible place (name, function).
- Nursing activities can be a source of strong stimulation (including pain) and may lead to an increase in the patient’s blood pressure and tachycardia - increasing analgosedation, or physician direct presence [30].
- **Patient's position** - half-high head of the bed (15-30°) in consultation with the ECMO physician, which contributes to fewer mechanical ventilation events (VAE-ventilator associated events), relieving the diaphragm and lower risk aspirations. Consequently, too high positioning of the patient may be associated with risk of bending the internal parts of the cannulas, bleeding from the site of introduction and/or formation of the pressure ulcers in the sacro-lumbar region [30].

*Changes of position should be made under supervision - one trained person delegated to protect cannulas.*

- Due to active anticoagulation, all actions, especially the ones effecting mucous membranes, require additional caution, including airway suctioning, oral and nasal care.
- Suctioning:
- *Catheter size:* diameter of the suction catheter should not exceed one half the inner diameter of the artificial airway in adults, providing an internal-to-external diameter ratio of 0.5 in adults,5,6 and 0.5-0.66 in infants and small children [40].
- *Strength (negative pressure applied)*: from -80 to -120 mmHg (from -10.7 to -15.9kPa)

the lowest effective value according to the principle.

- *Depth:* tube length closest to the connector + 8 cm; e.g. 28 cm + 8 cm = 36 cm; insert into tag 36, which means that the catheter is 1cm outside the tube; further standard execution.
- Oral care - according to standard protocols, as part of VAE prevention every 2-6 hours (according to regional protocols).
- Endotracheal tube fixation - soft attachments are preferred (commercial plastic handles may cause lesions earlier due to compression).
- If enteral feeding is planned, the stomach tube should be guided through the mouth. This should be placed before the start of any anticoagulant.
- Skin care - a haircut (clipper) or an electric shaver instead of a classic shave is suggested.
- Eye care - in accordance with the proper recommendations of the Society of Anaesthesiological and Intensive Care Nurses [34].
- Auscultation – The potential risk of personnel contamination and PPE used may make it impossible to use auscultation.

*Preferably, proper PPE should be available to assess the patient via a stethoscope. Should PPE not allow the use of a stethoscope, other methods to assess proper ETT positioning should be used. Supply special stethoscopes used specifically for rooms with contact precautions. These stethoscopes remain in the patient room for the duration of the patients stay.*

Assessment the position of the endotracheal tube should base on imaging methods.

- Use estimation methods to determine the depth immediately after intubation

e.g. based on patient height (Chula's formula: ETT depth = 0.1 * [height (cm)] + 4) [41].

- Position and protection of the cannulas – confirm position with the documentation.

*Daily measurement of the cannula’s is recommended to ensure cannula stability. By measuring from insertion site to end of cannula at connector and documenting this daily in patients electronic medical record.*

- In sterile conditions, daily routine inspection of the cannula insertion place/vascular accesses (with replacement of the dressing - if necessary or contamination). Assessment for: skin tone around the cannula, presence of secretions, blood; warming, swelling. If using foil or other dressings - replace them according to the manufacturer's instructions. If it is possible free part of cannulas and drains should be visible and uncovered.
- Assessment of the lower limb on the cannula introduction side - measurement of the leg circumference at a fixed place, pulse control on the peripheral artery distally to the cannulation access, temperature measurement on the distal part - continuous for R and L.
- If reperfusion cannula is used in VA ECMO, this line must remain visible (above dressing or below but transparent one [3]). Prevent its kinking and occlusion.
- In consultation with the therapeutic team - daily withdrawal of sedation and conducting a basic neurological evaluation [1,30].
- Control of the ECMO circuit - cannulas - protection against kinking, attachment to the patient's environment, such as bed sheet, observation for the presence of embolic material inside, line "chatter (cavitation)", assessment of clear difference in the color between oxygenated and non-oxygenated blood.
- Control of the oxygen supply source (gas flow [l/min] from the oxygen cylinder (fill level) or from the oxygen line (pay attention to the connection of the gas mixer to the line and oxygenator).
- ACT test (activated clotting time, coagulation time after activation, target value 180 through 240s) - every 2-4 hours in consultation with the attending physician.

**PHARMACOTHERAPY:**

- Analgesia and sedation - ECMO may change pharmacokinetics and pharmacodynamics of many medications (especially propofol, midazolam and opioids) due to an increase in the distribution volume. Additionally, some may be inactivated or sequestered in the circuit.
- It is suggested to prepare medicines for infusion pumps in the highest possible concentrations to minimize the frequency of syringe replacements, however current clinical condition should be assessed prior to concentrating infusions. Consultations with hospital pharmacists regarding the characteristics of the medicinal products (MPC) are necessary.
- It is suggested to use syringe pumps with the function of taking over the ending infusion by the second pump. If this is not possible, pay special attention to the smooth switching of the next infusion.

**MEDICAL DOCUMENTATION [2,37]:**

***ECMO and ABM parameters (acid-base management) - key information should be written on a whiteboard or windshield with a marker. A paper form of documentation is prepared in a clean area.***

● system of ECMO support,

● extracorporeal circuit (type, priming volume),

● size of cannulas, vascular accesses, distance at which cannulas are fixed,

● monitoring of technical parameters of extracorporeal support (console parameters, pressure before oxygenator (Pven), pressure after oxygenator (Part), (Pint), delta P, RPM, flow [L], battery charge status)

● monitoring parameters of mechanical lung ventilation parameters,

● monitoring of coagulation status and the doses of anticoagulants,

● routine inspections related to the safety of the technique carried out,

● technical interventions in case of complications,

● checklists.
