# Supplementary material for: Managing patients on extracorporeal membrane oxygenation support during the COVID-19 pandemic – a proposal for a nursing standard operating procedure
Source: BMC Nurs. 2021 Oct 30;20:214. doi: 10.1186/s12912-021-00736-7 (PMC8556777; doi:10.1186/s12912-021-00736-7)
Supplement: Supplementary file 3 — Additional file 3: Appendices C. Procedures with extracorporeal techniques. The normal font contains innovation suggestions, italic commentary as integrated part of discussion. [file 12912_2021_736_MOESM3_ESM.docx]

**PART C. PROCEDURES WITH EXTRACORPOREAL TECHNIQUES**

*The normal font contains innovation suggestions, italic commentary as integrated part of discussion.*

1. BEDSIDE ECMO INNITIATION/ECMO PATIENT ADMISSION

**A. PPE**

- See the general procedure for the use of personal protective equipment.

*For one-time entry into the “hot zone”, a barrier apron, gloves, goggles or a visor will be sufficient whereas a protective suit is recommended for 3 hours of continuous care periods* [7,8].

**B. PLACE:**

- Single or multi-person intensive care room. Required access to the power source (3-4 points and more) and 2 accesses to medical gases (oxygen, air).

*More space ensures work ergonomics. Because ECMO therapy takes up a lot of space, you may need to turn off every second ICU bed in multi person room.*

- Standard intensive care unit - electrically controlled bed with variable-pressure mattress, cardiac monitor (ECG, SpO2, invasive measurement of arterial pressure, temperature), stationary respirator, infusion pumps of at least 4 pieces (indicated program of the possibility of continuing the infusion in the second pump - smooth infusion taking), device for measuring coagulation of blood ACT (Hemochron (Instrumentation Laboratory, Bedford, MA, USA) or ACT (Helena Laboratories, Beaumont, TX, USA)- one for several ICU beds).
- Final equipment collection after final qualification of the patient by the implantation team.

**C. EQUIPMENT:**

- Standard equipment, i.e. Mayo table (for ECMO implantation) - they must be easily washable and disinfected.
- Ultrasound apparatus with linear, echocardiographic and convex transducers.

*All devices should be put in the patient's room. If transfer to another room or ward is necessary - mandatory detailed disinfection according to separate procedures. Ultrasound - the best tablet form with minimal number of knobs and spaces that are difficult to disinfect.*

- ECMO pump with oxygen cylinder, clamps (minimum 4) and emergency drive.

Heat exchanger - heater.

*Check that the gas line ends are compatible with the gas connectors in the central gas lines.*

*It is preferable to position the ventilator (for cannulation) on the left side of the patient, and the ECMO device on the right – either at the leg or a head of the bed depending on the ECMO configuration, the length of the cannulas, the currently performed procedure and the distance from the source of electricity and gases .*

*Ultrasound with TEE probe - when using a double-lumen cannula in the VV ECMO, for proper positioning, the use of TEE echocardiography is required. It is necessary to determine who will be responsible for the test during cannulation (PPE protection for this person). Standard disinfection of the probe and the entire ultrasound console (you can use a transparent, imposed protective film on the monitor and control panel). The latest ELSO guidelines (ELSO, 2020)* *do not recommend the routine use of a double-lumen cannula due to the need for additional staff to check the cannula position.*

*ELSO recommends that an additional ECMO device with a full system should be prepared for backup. A 60-day store of primed circuit system filled with a crystalloid solution is allowed* [5].

- Communication devices - mobile phones with a hands-free kit; wireless headphones; walkie-talkies; intercom.

*The use of a conventional telephone (mobile or landline) by personnel dressed in PPE without a hands-free kit is risky and not recommended.*

- A device for mechanical automatic chest compression (MACC).

*During respiratory failure despite optimal therapy, be prepared for cardiac arrest and resuscitation.*

**D. ADDITIONAL EQUIPMENT:**

- disposable set for the procedure - ECMO set (covers, aprons, ultrasound sleeves, clamps, sutures and materials for cannulas fixation);
- cannulas for cannulation (venous, arterial), connectors, introducers (100 cm and 150 cm) – in “hot zone”
- in the “cold zone”, a second set of cannulas and introducing sets should be prepared, including surgical set for cannulation; ECMO device with primed circuit.

*Use the common checklists or prepare individual for center to collect the necessary equipment*

*(Table 3).*

**E. TEAM:**

- 2 physicians for cannulation, (for double-lumen cannulation one with TEE skills) and 1 nurse – “hot zone” (Figure 1)
- 1 person for the ECMO circuit preparation and initiation – perfusionist or ECMO specialist; at least 1 nurse – in “cold zone”.

**F. DETAILED PROCEDURE DESCRIPTION:**

- **PLANNING:** roles and competences have been determined, efficient contact between “hot and cold zone” is ensured, at least one person on the cold side (having access to all necessary components of the system and therapy).
- The patient's position, cannulation sites and type of support must be defined.

*Check the invasive blood pressure measurement, position and attachment of the endotracheal tube, mechanical ventilation settings. The protected closed suction system, integrated into the respiratory system is recommended.*

*Central intravenous line (as many lumens as possible 3-4 minimum) - preferably on the LEFT side, alternatively consider changing the position.*

*Effective analgosedation has been provided.*

- **CANNULATION START:**

*Shearing of expected cannulation and spare places (groin - on both sides); consider giving a muscle relaxant (bolus) before cannulation; prepare a heparin bolus (50-100j/kg bw). Specify the size of the cannulas.*

***During the cannulation, a perfusionist/ECMO specialist prepares the system in the “cold zone”.***

*WARNING! In the case of heparinization contraindications and the case of therapy with Bivalirudin - a separate treatment protocol and therapy monitoring must be used.*

- **CANNULATION:**
  The TEAM performing cannula introduction, after surgical washing with 2% chlorhexidine of potential cannulation sites and placement of sterile cover (Figure 2-3). Cannulation followed by connection of the primed ECMO device to the system, then line fixation and eventually initiation of therapy.

*Note the depth of cannula insertion, fix them; apply dressings, e.g. Mepilex Border ® for cannulas and external dressings (foam or hydrocolloid). Fastening ECMO drains (careful not to damage) to the patient's environment - no kinking, no contact with the floor.*

**The blood samples or other specimens for laboratory analyses, particularly blood gases from the ECMO system should be transferred to POCT (point of care testing) in a previously developed way through a person from a “cold zone”.**

*Use clean bath basins in the „cold zone” where all the items needed to draw the blood are placed. Then place the container in the „clean” room (Ante room), and shut the door. The „hot zone” nurse takes the items from the bucket, draws the sample and places the sample inside a bag and seals it. Next this nurse places the bag back inside the bucket in the ante room, shut the door. The nurse in the „cold zone” gloves up and takes the bagged sample and places it into another clean bag (thus double bagging it), removes their gloves, washes hands and takes bagged sample to tube station.*

1. CIRCUIT CONTROL/COOPERATION WITH ECMO SPECIALIST
2. Short track – **„focused circuit check”** – daily or more often.

***CHECK:*** *Power connection, emergency power supply, gas connection, oxygenator, drains, cannulas.*

*Monitoring of line color before (correctly dark line) and after (light one) the oxygenator.*

*If the same dark color is present on the line - suspected oxygenator or gas supply failure.*

*In the event of an even bright line color - recirculation suspected during VV ECMO.*

1. INTRAHOSPITAL ECMO PATIENT TRANSPORTATION

A. PPE - See the general procedure for the use of personal protective equipment.

B. TEAM – minimum 2 people in the “hot zone” (preparation for transport and waiting in the “hot zone” for the patient after transportation); transport of 3-4 people.

*If it is possible plan intrahospital transports with full availability of the ECMO team in the hospital.*

C. EQUIPMENT - monitor and transport ventilator, syringe pumps, oxygen protection, ECMO emergency power supply.

*The preferred transport on the patient's bed eliminates the need for multiple shifting.*

***It is recommended to use existing checklists (checklist) and standard operating protocols (SOPs) commonly used or prepared individually for center special conditions and requirements.***

1. OTHER ACTIONS:
2. **CONNECTION OF CONTINUOUS RENAL REPLACEMENT THERAPY (CRRT) TO THE ECMO SYSTEM:**

A. PPE - See the general procedure for the its use.

B. Team - physician + nurse

C. General recommendations: **connecting the CRRT to the ECMO circuit.**

***Preferred connection: receive after the oxygenator, return before the oxygenator and after the pump.***

1. **PRONE POSITION IN ECMO:**

A. PPE - See the general procedure for the its use.

B. Team - min. 5 people

C. General recommendations: minimum one person protects ECMO cannulas and drains.

1. **REHABILITATION:**

A. PPE - See the general procedure for the its use.

B. Team - at least 2 people (1 person from ICU and 1 rehabilitation person)

C. General recommendations: one person protects ECMO cannulas and drains.

1. **CARDIOPULMONARY RESUSCITATION:**

A. PPE - See the general procedure for the its use.

B. Team - at least 2 people

C. General recommendations: Recommended use of automatic mechanical chest compression (MACC) devices. Prepare for cannulation conversion to VA.

***In VV ECMO – CPR with MACC, in VA ECMO CPR not necessary.***

1. **CANNULATION DURING CPR**

A. PPE – See the general procedure for the its use.

B. Team – 2 persons for CPR and 2 person for cannulation in “hot zone”, minimum one person in “cold zone” to prepare device and incidental intervensions..

C. Provide CPR with use of automatic mechanical chest compression (MACC) devices and support wentilation with intubation or suppraglottuc devices as soon as possible.

D. After confirmation of inclusion criteria for extracorporeal support cannulation team prepare procedure according to Part C.

E. Priority is to perform ultrasound guided percutaneous cannulation of femoral vessels. After ECMO START, STOP CPR. The distal shunt for femoral artery can be placed with delay.

1. **X-ray:**

A. PPE - See the general procedure for the its use.

B. Team - at least 1 person in the ICU (“hot zone”) + radiologist (“cold zone”).

C. General recommendations: radiation protection must be strictly observed:

• lead aprons/mobile lead screens - leaving in the “hot zone” or decontamination after use (if the patient is in isolation, then decontamination is recommended by choice, if the entire ward is a "COVID-19 ward" then the equipment can be left in “the COVID-19 area").

1. **BRONCHOSCOPY:**

A. PPE - See the general procedure for the its use.

B. Team - min. 2 people - nurse + physician

C. General recommendations: PPE - overalls, mask, goggles, visor.

1. **ECMO WEANING:**

A. PPE - See the general procedure for the its use.

B. Team - min 2 people - nurse (“hot zone”) + physician (“hot zone”)

+ perfusionist (“cold zone”)

C. General recommendations: long-term process
